# Maternal Vaccination and Neonatal Feeding Strategies Among Polish Women

**DOI:** 10.3390/vaccines13040376

**Published:** 2025-03-31

**Authors:** Jolanta Lis-Kuberka, Magdalena Orczyk-Pawiłowicz

**Affiliations:** Division of Chemistry and Immunochemistry, Department of Biochemistry and Immunochemistry, Wroclaw Medical University, M. Sklodowskiej-Curie 48/50, 50-369 Wroclaw, Poland

**Keywords:** maternal vaccination, breastfeeding, influenza and SARS-CoV-2 virus, infant’s protection, feeding strategy

## Abstract

**Background/Objectives**: Maternal vaccination and breastfeeding are important aspects of public health that should be recommended by medical staff caring for pregnant and postpartum women. We aimed to analyze factors affecting women’s likelihood of dual vaccination during pregnancy and their infant feeding strategies. **Methods**: A cross-sectional study was conducted with 953 Polish mothers. An online questionnaire was used and included questions on sociodemographic and obstetric variables, women’s attitudes towards COVID-19 and influenza vaccination, and breastfeeding practices. **Results**: COVID-19 vaccination was reported by 66.0%, influenza vaccination by 18.2%, and dual vaccination by 15.6% of Polish mothers. Increasing willingness to receive vaccines was significantly associated with older maternal age, lower BMI, living in urban areas with >100,000 residents, and high levels of knowledge regarding vaccination. No significant association between dual vaccination and neonatal feeding strategy was detected. The group of exclusively breastfeeding mothers, in comparison to formula- and mixed-feeding women, was characterized by having lower pre-pregnancy BMI and previous maternal experience. **Conclusions**: Rates of vaccination against seasonal influenza and dual (influenza and COVID-19) vaccination remain low among Polish mothers. The promotion of antenatal vaccination and reliable information about short- and long-term advantages related to breastfeeding are crucial to perinatal health care for the mother–infant dyad. Young, primiparous women who are overweight or obese should be targets of preventive programs focused on the health of the mother–infant dyad.

## 1. Introduction

Vaccination is key to primary health care and constitutes an important issue in public health. It has been reported that vaccination according to the standard recommendations allows individuals to avoid some infectious diseases and prevents approximately 2.5 million deaths yearly [1]. Health-enhancing behaviors like vaccination among mothers during pregnancy, in the perinatal period, and during lactation are particularly interesting. Antenatal vaccination induces a vaccine-specific immune response in the mothers, which includes the synthesis of specific IgG antibodies that not only protect women but also are transferred to the fetus, priming the developing fetus for postnatal life [2]. The transplacental passive transfer of IgG molecules is mediated by FcRn, which is localized in the placental syncytiotrophoblast, and this process is an important mechanism that protects the infant while his/her humoral response is inefficient [3]. The highest rate of maternal-antibody transport is observed at 28–32 weeks of gestation [2,4]. Maternal IgG antibodies transferred during the perinatal period provide passive immunity, and they represent a powerful tool for the protection of infant after birth, especially since newborns cannot produce IgG antibodies in the first months of their life [2,4,5,6,7]. In the months after birth, there is a decrease in maternal IgG levels in the neonatal circulation, which is the result of their catabolism (in the period of physiological hypogammaglobulinemia) and occurs between months 3 and 6 of life. This period overlaps with the endogenous synthesis of IgG by the infant, which begins approximately 15 weeks after birth [5,6,7].

Newborns and infants have immature immune systems and are susceptible to infections in the first years of life [8,9,10,11,12,13,14]. In the early stage of postnatal life, infections can cause morbidity and mortality in infants and are extremely dangerous for preterm neonates. Influenza virus, respiratory syncytial virus (RSV), rhinoviruses (RVs), and human parainfluenza viruses (HPIVs) are responsible for the majority of hospitalizations of infants suffering from lower respiratory tract infections [15,16,17,18,19,20]. Moreover, due to the immaturity of their immune systems, infants may be expected to be more susceptible to infection caused by the SARS-CoV-2 virus, which is associated with the occurrence of multi-inflammatory syndrome in neonates [21,22]. To protect against infection in infants and also in mothers, vaccinations during pregnancy are recommended. The Polish Society of Gynecologists and Obstetricians recommends, during pregnancy, the administration of vaccines containing inactivated pathogens, either whole or as fragments [23,24,25,26,27] The most common examples include influenza and pertussis vaccination, which represent significant breakthroughs in reducing perinatal mortality [28]. The COVID-19 pandemic introduced a third vaccine, the effectiveness and safety of which were ensured through rigorous testing criteria [23,28,29,30,31].

It has been reported that influenza/COVID-19 vaccination during pregnancy has positive outcomes for the mother–infant dyad, namely providing passive immunity to the infant before birth by transplacental transfer of vaccine-induced IgG antibodies and after delivery via the maternal milk, which contains specific antibodies (mainly S-IgA) that are transferred to the newborn/infant during breastfeeding [32,33,34,35,36,37]. Moreover, it has been reported that influenza/COVID-19 vaccination during pregnancy provides protection for women in the perinatal and postpartum period and prevents the virus’s transmission from mother to infant after delivery [38]. Zaman et al. [39] reported that transferred maternal IgG antibodies specific to influenza that were induced during pregnancy remained in infants’ circulation for 6 months and that a similarly high level of influenza vaccine-induced S-IgA antibodies was found in the maternal milk. Similarly, Edlow’s working group [40] reported that maternal COVID-19 vaccine-induced antibodies are detected in infants in the first year of life and that perinatal COVID-19 vaccination effectively reduces the risk of occurrence of COVID-19 among the youngest members of society, namely infants less than 6 months of age [40,41,42]. Working groups [43,44,45] demonstrated that pregnant women exhibit strong immune responses to COVID-19 vaccines, which allow them to achieve antibody levels comparable to those seen in non-pregnant women of reproductive age. Lopez et al. [40] highlighted that the persistence of antibodies in infants is associated with maternal vaccine timing, placental Fc-receptor binding capabilities, and antibody subclass. Moreover, it should be pointed out that vaccination of women should be performed even for individuals who have been infected in the past because it has been reported that higher transfer of virus-specific IgG from mother to fetus is observed with infection plus vaccination [46]. Maternal vaccination plays an essential role in providing neonates with protection because influenza and COVID-19 vaccinations are not available for infants under 6 months of age [34,35,47,48,49,50,51].

Even though perinatal vaccinations are recommended for women during pregnancy and during breastfeeding, data regarding the impact of vaccination on the choice of feeding strategy for neonates are limited [52,53]. It is well known that breastfeeding offers many health benefits to newborns and infants. As reported by Ladomenou et al. [54], independent of neonatal gestational age and birth weight and also the type of delivery, the frequency and duration of breastfeeding are among the main factors affecting the valuable effect of human milk. According to the World Health Organization (WHO) [55], breastfeeding exclusively for six months is considered the gold standard and leads to lower risk of infections (e.g., respiratory infection and acute otitis media) and hospital admissions in comparison to not breastfeeding [54]. Another working group [56] found that the odds of having had a disease with fever in the last two weeks were 66% lower among infants who were exclusively breastfed than among non-exclusively breastfed neonates. Moreover, exclusively breastfed infants have lower odds of having a disease with cough and diarrhea than non-exclusively breastfed infants. In light of this evidence, breastfeeding as a neonatal feeding strategy in the first months of the infant’s life should be especially recommended by obstetricians and nurses taking care of women in the perinatal period. Exclusive breastfeeding for the first half year of an infant’s life, with continued breastfeeding up to 2 years of age or beyond, is recommended by the WHO as the gold standard for the nutrition of newborns and infants [57] and yields substantial benefits for both infant and mother. In Poland, the breastfeeding rate reported by mothers at the beginning of lactation is quite high (97–99.4%) [58,59,60,61,62]. However, women who decide to continue exclusive breastfeeding up to 6 months represent only 4–22.4% of mothers [59]. It has been reported [62] that the factors most frequently associated with a mother’s decision to discontinue breastfeeding are too few lactation consultants, problems with breastfeeding after returning to work, and negative attitudes towards breastfeeding in public places [62]. Moreover, it has been reported that Polish women have moderate knowledge of breastfeeding benefits and poor knowledge about correct breastfeeding attachment and positioning techniques [63,64,65,66].

Artzi-Medvedik et al. [52] identified an association between influenza vaccination during pregnancy and breastfeeding for at least three months in a population of women from the United States. On the other hand, Weston et al. [53], based on research including 18 breastfeeding mothers who received COVID-19 vaccination, highlighted that an individual woman’s habitus has an impact on her knowledge level, attitudes, and beliefs and interacts with options for infant feeding and health-promoting behaviors such as vaccination. So far, the impact of dual vaccination on breastfeeding as a neonatal feeding strategy has not been analyzed. In light of the above, the primary aim of this study was to evaluate whether mothers’ influenza and/or COVID-19 vaccination status was associated with their choice of infant feeding strategy in the obstetric population. The secondary objectives of this study included evaluating the knowledge level regarding maternal vaccination and the identification of factors associated with perinatal vaccination. The second part of the analysis involves the evaluation of variables associated with the choice of breastfeeding as a strategy for infant feeding. Finally, the relationship between health-enhancing behaviors such as maternal vaccination and breastfeeding was assessed. The sociodemographic and obstetric variables identified in this study will help select the most vulnerable groups of Polish women, those who should be the targets of preventive programs regarding the health of the mother–infant dyad.

## 2. Materials and Methods

### 2.1. Study Design and Participants

**Figure 1:**
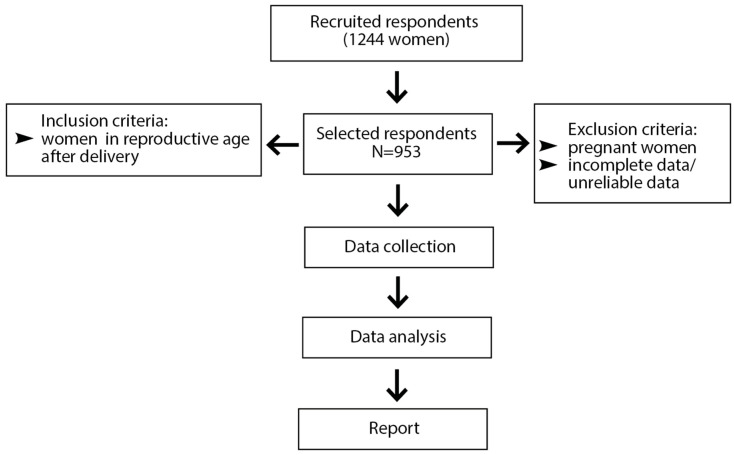
Flow chart for respondents’ inclusion in the study.

An anonymous survey was used to assess rates of influenza vaccination, influenza/COVID-19 (dual) vaccination, and breastfeeding among Polish women. The research was approved by the Ethics Committee at Wroclaw Medical University (No. KB-356/21). The survey was composed of the following sections: (1) maternal sociodemographic data, (2) obstetric variables, and (3) knowledge regarding maternal vaccination and influenza and COVID-19 vaccination among Polish mothers. The primary version of the questionnaire was subjected to pilot testing. Comments and suggestions from five mothers were included in the development of the final version of the online survey. Cronbach’s alpha was used for the reliability assessment of the questionnaire, and it ranged from 0.30 to 0.62. The survey was administered online (with respondents recruited via Facebook), and it targeted women who were pregnant or had recently delivered an infant. This study is a secondary analysis of data collected from previous research [67]. The total number of women recruited for the study was 1244. However, because the analysis focused on health-promoting behaviors and also examined infant feeding strategies, the analysis excluded pregnant women. Incomplete and unreliable data were excluded from the analysis. The final dataset included 953 postpartum mothers (Figure 1). This was a cross-sectional (observational) study.

### 2.2. Data Source and Study Size

The survey was conducted from 15 November 2021 to 31 March 2022 among Polish women. The survey was administered using Google Forms, and potential participants were recruited for the study through parenting communities via Facebook. The data regarding COVID-19 vaccination status were expanded to include the information about influenza vaccination and data regarding feeding strategies for infants. Finally, based on sociodemographic and obstetrical data, as well as data on knowledge about vaccination, we evaluated the association of vaccination against influenza and COVID-19 with breastfeeding among Polish mothers.

To calculate the sample size (calculated using TIBCO STATISTICA ver. 13.3, StatSoft, Inc., Tulsa, OK, USA), we assumed a 95% confidence level (α error of 0.5%) and a 5% loss rate. Based on the total number of births in 2021/2022 (318,000) and the level of COVID-19 vaccination of the Polish population (66.0%), the minimum required sample size was 368 subjects.

### 2.3. Quantitative and Qualitative Variables

Data on maternal age and anthropometric variables such as body weight and height were provided by the participants of the research and collected as quantitative data. In the following step of the performed analysis, maternal age was categorized into ranges of 18–25, 26–34, and ≥35 years, while pre-pregnancy body mass index (BMI) (calculated based on the data provided by respondents and classified according to the WHO guidelines [68]) data were categorized as follows: underweight (<18.5 kg/m^2^), normal weight (18.50–24.9 kg/m^2^), overweight (25.0–29.9 kg/m^2^), and obese (≥30 kg/m^2^).

The data regarding education level (vocational and primary, high school, university), marital status (single parent and divorced, cohabiting, married), residence (rural area, urban <10,000 residents, urban >100,000 residents, urban 10,000–100,000 residents), obstetric data (mode of delivery: vaginal birth, elective cesarean section, emergency cesarean section), maternal experience (no maternal experience, 1 child, 2 children, ≥3 children), and neonatal feeding strategies (breastfeeding, mixed feeding, formula feeding) were classified as qualitative variables.

The assessment of the mother’s knowledge level concerning perinatal vaccination was estimated based on the following questions: “Can the SARS-CoV-2 virus be transmitted via human milk?”; “Can breastfeeding mothers who received COVID-19 vaccination reduce the risk of this disease in their child?”; “Can COVID-19 vaccine-induced humoral immunity be transferred by the placenta to the fetus?”; “Can COVID-19 vaccine-induced humoral immunity be transferred to infants via breast milk?”.

The respondents could answer “Yes”, “No”, or “I do not know”. The maternal knowledge score was computed as follows: a correct response scored +1, no answer or “I do not know” scored 0, and an incorrect response scored −1. The details concerning the assessment of maternal knowledge levels were adapted from Ramli et al. [69]. The mother’s knowledge level regarding perinatal vaccination was categorized as follows: score 4 out of 4 (100%)—detailed knowledge, score 2 or 3 out of 4 (50–75%)—moderate knowledge, and score lower than 2 out of 4 (<50%)—poor knowledge.

### 2.4. Statistical Analysis

All data, including continuous and nominal (categorical) variables, were analyzed using TIBCO STATISTICA, version 13.3 (StatSoft, Inc., Tulsa, OK, USA). Means and standard deviations (the mean ± SD) and median and 25–75% interquartile range were reported to describe continuous variables (age and BMI of respondents) and frequencies and percentages (% (n/N)) were reported to describe categorical variables. To compare groups, the chi-square test was used. To test the research questions, multiple comparison tests (Bonferroni) for related samples were used.

The variables (age, BMI, residence, education, marital status, and mother’s level of knowledge about maternal vaccination) were selected based on the result of the chi-square test and then subjected to univariate analysis.

Multivariate logistic regression was used to determine which of the predictors of positive health-related behaviors, such as breastfeeding, among Polish mothers yielded *p* < 0.05 using the odds ratio (OR). In the initial model, considering a significance level of 0.1, the following variables were selected for inclusion in the multivariate logistic regression model: BMI, level of knowledge about maternal vaccination, influenza vaccination, and dual vaccination. An analysis of linearity and additivity of the relationship between variables was performed. As a method for scoring and selecting a model, the best subset (all effects) was used. The confidence level was set at 95%, with statistical significance defined as *p* < 0.05. For multivariate logistic regression, the following parameters were obtained: β coefficient *p* < 0.05, and for Hosmer–Lemeshow’s test, *p* = 0.68.

## 3. Results

### 3.1. Sociodemographic and Obstetric Data of Respondents

The mean age of the respondents was 31.0 ± 4.4 years, with a range of 18–46 years. Most of the participants (623/953; 65.4%) had a normal pre-pregnancy BMI, and women who were underweight, overweight, or obese constituted 6.4% (61/953), 18.6% (177/953) and 9.7% (92/953) of the sample, respectively. Mothers living in urban areas with a population >100,000 (460/953; 48.3%) constituted the majority of the group, while 20.6% of respondents reported living in cities with a population of 10,000–100,000 residents and 24.2% of women reported living in a rural area. Higher education was reported by 79.2% of participants (755/953). Married women constituted 82.1% of participants (782/953), while single parenthood and divorced status were reported by 1.9% of the participants (18/953) (Table 1).

In the analyzed obstetric population, the majority, 55.8% (532/953), reported vaginal delivery (Table 1). Mothers with prior maternal experience constituted more than 90.3% (861/953) of respondents (Table 1). Further, 80.2% of respondents reported breastfeeding as a neonatal feeding approach, while formula and mixed feeding were reported by 9.0% and 10.8%, respectively. Additionally, 66% of respondents reported COVID-19 vaccination, 18.2% reported influenza vaccination, and only 15.6% reported dual vaccination.

### 3.2. Sociodemographic and Obstetric Data of Respondents in Relation to Vaccination Status

In the present study, 66.0% (623/953) and 18.2% (173/953) of mothers reported that they had received the COVID-19 and influenza vaccines, respectively. Dual vaccination was reported by 15.6% (149/953) of respondents (Table 1).

The analysis of sociodemographic data in relation to maternal vaccination status showed that the women most likely to have been vaccinated were 26−34 years old, had higher education, and lived in urban areas with > 100,000 residents. For dual vaccination and COVID-19 vaccination, significant differences in categorical data such as age, residence, and education were noted. In contrast, for mothers who had received the influenza vaccine, significant differences in pre-pregnancy BMI and residence were recorded (Table 2). The Bonferroni multiple-comparisons test was performed to determine which variables significantly differed from each other. The results are displayed in Supplementary Materials Table S1. The results showed that rates of dual/COVID-19 vaccination significantly differed with respect to age in the following comparisons: 18–25 vs. 26–34 (*p*-value from 0.00004 to 0.04) and 18–25 vs. ≥35 (*p*-value from 0.00002 to 0.04). Moreover, rates of influenza vaccination and/or COVID-19 vaccination significantly differed with respect to place of residence, specifically for urban areas with >100,000 residents vs. <10,000 residents (*p* < 0.02 for dual and *p* < 0.008 for COVID-19 vaccination), urban areas with >100,000 vs. <10,000 residents (*p* < 0.0004 for COVID-19 vaccination), and urban areas with >100,000 residents vs. rural areas (*p* < 0.0008 for dual and *p* < 0.000001 for COVID-19 vaccination). Significant differences were also seen with respect to educational level in the comparison of high school vs. university education (*p* < 0.007 for dual vaccination and *p* < 0.00001 for COVID-19 vaccination).

Regardless of the type of vaccination, significant differences in level of knowledge about maternal vaccination (dual vaccination *p* < 0.0008, COVID-19 vaccination *p* < 0.0002, and influenza vaccination *p* < 0.00002) were noted among mothers (Table 2). On the other hand, the analysis of obstetric data in relation to vaccination status showed no significant differences in type of delivery (*p* < 0.9) (Table 2). The results of the Bonferroni multiple-comparisons test showed that rates of influenza vaccination and/or COVID-19 vaccination significantly differed with respect to women’s knowledge levels in the following comparisons: detailed vs. poor knowledge (*p*-value from <0.00001 to 0.0005) and moderate vs. poor knowledge (*p*-value from <0.00001 to 0.0006) (Supplementary Materials Table S1).

### 3.3. Sociodemographic and Obstetric Data of Respondents in Relation to Neonatal Feeding Patterns

In the present study, 80.2% (764/953) of the mothers reported breastfeeding, while the mixed and formula-feeding strategies were reported by 10.8% (103/953) and 9.0% (86/953), respectively (Table 1). The stratified analysis of sociodemographic and obstetric data of respondents in relation to neonatal feeding revealed significant differences for the following variables: maternal pre-pregnancy BMI (*p* < 0.006), prior experience of motherhood (*p* < 0.0001), and level of knowledge about maternal vaccination (*p* < 0.03) (Table 3). In contrast, the respondents’ age, place of residence, education, marital status, and obstetric variables, and status with regard to COVID-19 vaccination, influenza vaccination, or dual vaccination did not show significant differences between mothers in relation to neonatal feeding strategy (Table 3).

The Bonferroni multiple-comparisons test was performed to determine which variables significantly differed from each other. The results are displayed in Supplementary Materials Table S2. The results showed that neonatal feeding strategy significantly differed with respect to BMI in the comparisons normal weight vs. overweight (*p* < 0.002) and overweight vs. obese (*p* < 0.003), and with respect to maternal experience in the following comparisons: no maternal experience (0) vs. 1 child (*p* < 0.0002), no maternal experience (0) vs. 2 children (*p* < 0.02), no maternal experience (0) vs. ≥ 3 children (*p* < 0.012).

### 3.4. Respondents’ Sociodemographics and Obstetric Data in Relation to Maternal Experience

In the present study, 90.3% (861/953) of the women reported previous maternal experience. The percentages of women with one, two, and three or more offspring were 50.7% (483/953), 28.1% (268/953), and 11.5% (110/953), respectively (Table 1). The stratified analysis of respondents’ sociodemographic and obstetric data in relation to maternal experience revealed significant differences among analyzed groups for the following variables: age (*p* < 0.0001), place of residence (*p* < 0.006), education (*p* < 0.0001), marital status (*p* < 0.008), and mode of delivery (*p* < 0.0001) (Table 4). In contrast, the respondents’ pre-pregnancy BMIs, levels of knowledge about maternal vaccination, and rates of COVID-19 vaccination, influenza vaccination, or dual vaccination did not show significant differences among analyzed subgroups in relation to maternal experience (Table 4). 

The Bonferroni multiple-comparisons test was performed to determine which variables significantly differed from each other. The results are displayed in Supplementary Materials Table S3. The results showed that maternal experience significantly differed with respect to age in the comparisons between those aged 18–25 vs. 26–34 (*p* < 0.0004), 18–25 vs. ≥35 (*p* < 0.00001), and 26–34 vs. ≥35 (*p* < 0.00001); with respect to place of residence in the comparison >100,000 residents vs. rural areas (*p* < 0.042); with respect to education in the comparisons vocational/primary vs. university (*p* < 0.048) and high school vs. university (*p* < 0.00005); and with respect to mode of delivery in all analyzed categories (*p*-values from <0.00001 to 0.01) (Supplementary Materials Table S3).

### 3.5. Associations Between Sociodemographic Variables and Dual Vaccination Among Mothers

The multivariate logistic analysis showed that predictors of dual vaccination include the mother’s age, BMI, place of residence, and knowledge level regarding maternal vaccination (Table 5).

Respondents with BMIs categorizing them as obese significantly (*p* < 0.009) less often decided to receive dual vaccination (OR = 0.34; 95% CI = 0.15–0.77). Similarly, a lower rate of dual vaccination was observed for respondents from rural areas and medium-sized urban areas (10,00–100,000 residents) (OR = 0.52; 95% CI = 0.31–0.88, *p* = 0.01 and OR = 0.52; 95% CI = 0.31–0.87, respectively, *p* = 0.02). A low knowledge level regarding maternal vaccination was associated with not being vaccinated (OR = 0.31; 95% CI = 0.19–0.45, *p* < 0.001) (Table 5).

### 3.6. Associations Between Sociodemographic Variables, Health-Enhancing Behaviors, and Neonatal Feeding Strategy

For the analyzed group of mothers, the multivariate analysis identified three potential predictors of choosing breastfeeding for neonatal feeding: BMI, maternal experience, and knowledge level regarding maternal vaccination.

The respondents with BMIs categorizing them as obese significantly (*p* < 0.001) less often choose breastfeeding as a method of neonatal feeding (OR = 0.40; 95% CI = 0.24–0.65). Similarly, a low level of knowledge regarding maternal vaccination was associated with a lower rate of breastfeeding (OR = 0.63; 95% CI = 0.44–0.90, *p* < 0.02). Additionally, a lack of prior experience of motherhood was associated with a lower rate of breastfeeding (OR = 0.41; 95% CI = 0.25–0.67 *p* < 0.0001).

Influenza and/or COVID-19 vaccination were not significantly associated with breastfeeding as a strategy for feeding (Table 6).

## 4. Discussion

Maternal vaccination and breastfeeding are important aspects of public health that should be highlighted by medical staff caring for pregnant and postpartum women. A working group [28] has pointed out that pregnancy is associated with increased severity of some infectious diseases. Given the incidence of influenza/COVID-19 in pregnancy and the neonatal morbidity due to infection in early postnatal life, maternal vaccination in pregnancy is recommended [28,42]. The mother and child form a unique relationship, called the mother−infant dyad, and in light of this, health-enhancing behaviors are crucial for the short- and long-term health of both. This study sheds light on mothers’ levels of knowledge regarding vaccine-induced humoral immunity for mother−infant dyads and identifies variables associated with choosing breastfeeding as an infant feeding strategy.

In Poland, according to the guidelines of the Polish Society of Gynecologists and Obstetricians, influenza/COVID-19 vaccination is free of charge for pregnant women, who are considered as a priority category [23,26,27,70,71]. Despite recommendations by obstetricians and the well-established status of influenza vaccination, in the present study, less than 20% of respondents had decided on influenza vaccination; by contrast, in high-risk populations, the optimal vaccination coverage target is 95% [72]. The present data are in line with those in previous reports [73,74,75], which showed that influenza vaccination coverage remains low, especially in low- and middle-income countries [76]. A working group [73] has found that in many developed countries, the influenza vaccination rates are low due to concerns about vaccine safety, as well as due to individuals downplaying the disease and underestimating the advantage of vaccination. In contrast, COVID-19 vaccination was reported by 66.0% of respondents (66.5% in the breastfeeding group, 67.0% in the mixed-feeding group, and 60.5% in the formula-feeding group). Dual vaccination was recorded for only 15.6% of respondents (16.6% in the breastfeeding group, 12.6% in the mixed-feeding group, and 10.5% in the formula-feeding group). The stark differences in vaccination acceptance may be the result of a low level of knowledge among mothers about the advantages of perinatal vaccination, insufficient promotion of influenza vaccination by public institutions in the country, and inadequate education of women of reproductive age. Currently, vaccinations are the most common method used to prevent infectious diseases. Therefore, public-health programs should be adapted to the needs of each population by identifying key factors influencing the avoidance of vaccination by pregnant women and mothers. This study showed that lessons learned from the COVID-19 pandemic do not impact maternal decisions regarding perinatal vaccination. Although COVID-19 vaccination was reported by 66% of respondents, the rates of influenza vaccination were low among the analyzed groups. Therefore, the successful promotion of maternal vaccinations should be focused on providing reliable information to women of reproductive age in areas with negative outcomes of disease and the possibility of avoiding them by vaccination.

In this study, we identified age, BMI, place of residence, and knowledge level regarding maternal vaccination as factors significantly associated with willingness to be vaccinated. The significant differences between those aged 18–25 vs. 26–34 and those aged 18–25 vs. ≥35 and between those residing in urban areas with >10,0000 residents and those residing in rural areas (Table 3) suggest that young women living outside urban areas should be a target of campaigns promoting maternal vaccinations. Our data align with those of previous reports [77,78] demonstrating that age, rural residency, and chronic diseases were associated with avoiding influenza/COVID-19 vaccination. Data demonstrating the impact of obesity/BMI of respondents on the likelihood of accepting COVID-19 vaccination are limited. According to Kessy et al. [79], the likelihood of accepting COVID-19 vaccination was significantly associated with respondents’ age, BMI, education, and residency, and these data are in line with our results. The authors pointed out that high BMI and comorbidities may influence an individual’s inclination to accept vaccination [79]. Townsend et al. [80] noted that vaccine hesitancy specifically among obese individuals is concerning for several reasons, including prevalent weight bias, which hinders their engagement in the healthcare system, marginalizes people with obesity, and reduces use of recommended preventive care. Additionally, high BMI is associated with lower COVID-19 vaccine effectiveness and a reduced immune response to vaccination [81,82]. The authors identified, as the main reasons, the poorer innate and adaptive immune responses, as well as the associated impaired T-cell response, associated with obesity and suggested the need for a booster to enhance protection [82].

It is crucial for dedicated programs to highlight that mothers, by accepting vaccines during the perinatal period, may contribute to the well-being of their infants. As the experience of the COVID-19 pandemic showed, a relatively high COVID-19 vaccination rate (66%) can be achieved, whereas double vaccination was reported by only 15.6% of respondents in the study group. These rates are dramatically low and require urgent corrective actions. As reported previously [67,75], the most common reason for avoiding COVID-19 and influenza vaccination was possible overall post-vaccination complications.

Moreover, in this study, we observed that breastfeeding mothers, in comparison to formula- and mixed-feeding women, were more likely to have lower pre-pregnancy BMI and maternal experience; this finding is in line with data presented by other authors [83]. It has been reported that mothers with higher BMI are less likely to develop successful breastfeeding in comparison to normal-weight women [83,84]. The main reasons for the lower rate of breastfeeding in the obstetrical population with overweight or obesity are delayed lactogenesis, failure to initiate breastfeeding, and/or exclusive breastfeeding only during hospital admission [83,84].

The impact of prior experience of motherhood on rates of breastfeeding was probably the net result of previous positive experience with breastfeeding practices, and this supposition is consistent with previous reports [85,86]. A working group [87] has reported that a mother’s education level plays a pivotal role in the choice of infant feeding strategy. Although we did not observe a significant association between education level and mothers’ decisions regarding neonatal feeding (Table 3), we noted that this variable was significantly related to prior experience of motherhood (Table 4). Wako et al. [88] reported that maternal education has an impact on socioeconomic status, which can be associated with health-enhancing behavior and also improve understanding of the short- and long-term health benefits of breastfeeding for the mother–infant dyad. Our results are convergent with these findings, and we suggest that women with higher education and maternal experience might have higher motivation to acquire knowledge in areas relevant to improving maternal and infant health. Moreover, the level of knowledge regarding maternal vaccination was significantly different between breastfeeding mothers and women who decided on formula or mixed feeding, and these observed data are in line with the findings of other authors [89]. They [89] found that mothers’ knowledge regarding infants’ nutrition is not sufficient and that complementary feeding practices require corrective action. In contrast to the results of previous research [90,91], in this study, we did not observe significant differences in maternal age between the analyzed groups. Additionally, no significant difference in vaccination status was found between breastfeeding mothers and women who used formula or mixed feeding.

The protective effect of maternal vaccination as a strategy to reduce susceptibility to infection in newborns and infants is well established [37,92,93,94]. As shown in this study, the promotion of COVID-19 vaccination among the Polish population of women of reproductive age translates into a high rate of vaccination among respondents. At the same time, in the years 2021–2022, influenza vaccination was less strongly promoted and the rate of vaccination in the same population was significantly lower. Our data indicated that campaigns promoting the safety of vaccinations and the broad range of benefits for the mother and infants are needed. The linking of the positive short- and long-term advantages of maternal vaccination might translate to a higher rate of annual vaccination. Previous studies [37,95,96] have reported that providing reliable information about the benefits of immunization effectively promotes maternal vaccination. In our study, we found that perinatal vaccination in mothers was not associated with breastfeeding as a strategy for infant feeding. Therefore, independent of the promotional campaigns regarding maternal vaccination, women of reproductive age should be informed about the valuable effect of maternal milk and the advantages of breastfeeding for both infants and mothers. The data indicate that the willingness of lactating women to get the COVID-19 vaccine was high, and factors associated with receiving vaccination in the obstetric population include medical history, belief that the COVID-19 vaccine is safe during breastfeeding, and the country of residence [97,98]. On the other hand, our study and the results of another group [99] are consistent and show that willingness to get the influenza vaccine in the obstetric population is low. There is a great need for urgent implementation of corrective actions to enhance vaccination efforts. Our study showed that in the Polish obstetric population, special education programs should be focused especially on young, primiparous women with overweight and obesity (Table 3, Table 5 and Table S2). We believe that reliable information regarding maternal vaccination and health enhancing-behaviors, including breastfeeding practices, improve general health literacy.

### Strength and Limitations

The undeniable strength of the present study is that it fills the knowledge gap regarding maternal vaccination among women of reproductive age. Another strength is the identification of sociodemographic predictors of avoiding dual vaccination. Based on these, general health-promoting programs aimed at pregnant women and mothers may be created. Additionally, our results offer a valuable and useful perspective on which factors are pivotal for shaping health-enhancing behaviors. This may translate into the well-being, in first months of postnatal life, of future generations.

We should acknowledge some limitations of our studies. First, attitudes regarding maternal vaccination among women might have changed during the data collection period. Future studies should explore the temporal dynamics of willingness to receive maternal vaccinations among women of reproductive age. Second, the study included 953 mothers who were recruited for the study through local parenting groups such as Facebook. Hence, the percentage of breastfeeding reported here does not represent the rate of neonatal breastfeeding in the general Polish population. In this study, we focused on the impact of some sociodemographic factors, while we did not consider individual-level psychological variables, which also play an important role in attitudes to vaccination. Moreover, continuation of research in this area might fill the knowledge gap concerning dual vaccination among women of reproductive age, and investigating the interplay between individual, sociodemographic, and sociocultural (e.g., social norms, peer influence, community support) variables might enable an in-depth and multi-faceted analysis of the problem of vaccination among pregnant and breastfeeding mothers. This research was exploratory, and some covariables were not accounted for. These included the rates of pertussis vaccination, maternal socio-economic status, obstetric interventions, and reasons for avoiding vaccination. All these factors have been shown to influence breastfeeding practices [76,100,101,102,103].

## 5. Conclusions

In the population of Polish mothers, primiparous women of reproductive age should be given special care, which includes knowledge regarding maternal vaccination. Older maternal age, lower BMI, living in urban areas with >100,000 residents, and a high level of knowledge regarding prenatal vaccination were identified as the main factors associated with increasing mothers’ willingness to be vaccinated. In the future, a pivotal strategy will be to generate specific antibodies in pregnant women that will have protective benefits for the infant. Currently, research is being conducted to develop new vaccines for immunizing mothers, namely vaccines against cytomegalovirus (currently in phase II research) and group B streptococcus to prevent late infections in newborns (phase II research) [28,104,105]. As demonstrated in this study, the influenza vaccination and/or COVID-19 vaccination was not associated with breastfeeding as a neonatal feeding strategy. In light of this, independent of the mother’s vaccination status, women should be informed about the unique advantages of breastfeeding such as preventing infections, modulating gut microbiota, and supporting mucosal immunity in neonates and infants. The multipronged approach of prenatal education and support should make women aware of the difficulties that can be faced by women after giving birth, especially by those who are overweight or obese (i.e., delayed lactogenesis, latching issues). Proper education during pregnancy, support from medical-care staff including healthcare workers and midwives, assistance from lactation consultants, and regular follow-up appointments to monitor the neonate’s growth and the mother’s breastfeeding experience can help identify and address issues early, ultimately improving outcomes for the mother–infant dyad.

## Figures and Tables

**Table 1:** General characteristics of participants.

Data		n/N	%
Age (years)	Mean ± SD Median (interquartile range 25–75%)	31.0 ± 4.4 31.0 (28.0–34.0)	
	18–25	92/953	9.7
	26–34	649/953	68.1
	≥35	212/953	22.2
Pre-pregnancy BMI, kg/m^2^	Mean ± SD Median (interquartile range 25–75%)	23.5 ± 4.3 22.5 (20.4–25.5)	
	Underweight (<18.5)	61/953	6.4
	Normal weight (18.5–24.9)	623/953	65.4
	Overweight (25–29.9)	177/953	18.6
	Obesity (≥30)	92/953	9.7
Residence	Urban, >100,000 residents	460/953	48.3
	Urban, 10,000–100,000 residents	196/953	20.6
	Urban, <10,000 residents	66/953	6.9
	Rural	231/953	24.2
Education	Vocational and primary	23/953	2.4
	High school	175/953	18.4
	University	755/953	79.2
Marital status	Married	782/953	82.1
	Cohabiting	153/953	16.1
	Single parent and divorced	18/953	1.9
Mode of delivery	Vaginal birth	532/953	55.8
	Elective caesarean section	219/953	23.0
	Emergency caesarean section	202/953	21.2
Maternal experience	0	92/953	9.7
	1	483/953	50.7
	2	268/953	28.1
	≥3	110/953	11.5
Neonatal feeding strategy	Breastfeeding	764/953	80.2
	Mixed feeding	103/953	10.8
	Formula feeding	86/953	9.0
COVID-19 vaccination	Yes	629/953	66.0
	No	324/953	34.0
Influenza vaccination	Yes	173/953	18.2
	No	780/953	81.8
Dual vaccination	Yes	149/953	15.6
	No	804/953	84.4

The table shows the percentage of respondents in the given subgroup (n) in relation to all respondents (N) for whom the specific information was available.

**Table 2:** Sociodemographics and health-enhancing behaviors of respondents in relation to maternal vaccination status.

Data		Dual Vaccination		Chi^2^ *p*-Value	COVID-19 Vaccination		Chi^2^ *p*-Value	Influenza Vaccination		Chi^2^ *p*-Value
		Yes N = 149	No N = 804		Yes N = 629	No N = 324		Yes N = 173	No N = 780	
Age (years)	Mean ± SD Median (interquartile range 25–75%)	32.01 ± 3.94 32.0 (29.0–34.0)	30.8 ± 4.4 30.0 (28.0–34.0)		31.5 ± 4.2 31.0 (29.0–34.0)	30.0 ± 4.6 29.0 (26.0–33.0)		31.6 ± 4.2 31.0 (29.0–34.0)	30.8 ± 4.4 30.0 (28.0–34.0)	
	18–25	6.3% (6/953)	9.1% (87/953)	Chi^2^ = 6.7 *p* < 0.04	4.3% (41/953)	5.5% (52/953)	Chi^2^ = 23.1 *p* < 0.00009	1.2% (11/953)	8.6% (82/953)	Chi^2^ = 3.0 *p* < 0.3
	26–34	11.1% (106/953)	56.9% (542/953)		45.9% (437/953)	22.1% (211/953)		12.6% (120/953)	55.4% (528/953)	
	≥35	3.9% (37/953)	18.4% (175/953)		15.8% (151/953)	6.4% (61/953)		4.4% (42/953)	17.8% (170/953)	
Pre-pregnancy BMI, kg/m^2^	Mean ± SD Median (interquartile range25–75%)	22.59 ± 3.75 21.8 (20.2–24.1)	23.6 ± 4.3 22.7 (20.5–25.7)		23.7 ± 4.5 22.7 (20.5–25.7)	23.1 ± 3.9 22.5 (20.3–25.1)		22.6 ± 3.7 22.0 (20.2–24.2)	23.6 ± 4.4 22.8 (20.5–25.7)	
	Underweight (<18.5)	1.2% (11/953)	5.6% (53/953)	Chi^2^ = 8.0 *p* < 0.05	4.4% (42/953)	2.3% (22/953)	Chi^2^ = 0.2 *p* < 0.9	1.4% (13/953)	5.4% (51/953)	Chi^2^ = 8.6 *p* < 0.04
	Normal weight (18.5–24.9)	11.5% (110/953)	53.9% (514/953)		42.6% (406/953)	22.9% (218/953)		13.3% (127/953)	52.2% (497/953)	
	Overweight (25–29.9)	2.2% (21/953)	16.1% (153/953)		11.9% (113/953)	6.4% (61/953)		2.5% (24/953)	15.7% (150/953)	
	Obesity (≥30)	0.7% (7/953)	8.8% (84/953)		9.3% (89/953)	2.4% (23/953)		0.9% (9/953)	8.6% (82/953)	
Residence	Urban, above 100,000 residents	10.3% (98/953)	40.0% (362/953)	Chi^2^ = 21.9 *p* < 0.00007	36.9% (352/953)	11.3% (108/953)	Chi^2^ = 21.5 *p* < 0.0003	10.7% (102/953)	37.6% (358/953)	Chi^2^ = 10.3 *p* < 0.02
	Urban, 10,000–100,000 residents	2.3% (22/953)	18.3% (174/953)		13.2% (126/953)	7.3% (70/953)		3.1% (30/953)	17.4% (166/953)	
	Urban, <10,000 residents	7.3% (7/953)	6.2% (59/953)		3.7% (35/953)	3.2% (31/953)		1.2% (11/953)	5.8% (55/953)	
	Rural	2.3% (22/953)	21.9% (209/953)		12.2% (116/953)	12.1% (115/953)		3.1% (30/953)	21.1% (201/953)	
Education	Vocational and primary	4.2% (4/953)	2.0% (19/953)	Chi^2^ = 9.5 *p* < 0.009	1.0% (10/953)	1.4% (13/953)	Chi^2^ = 36.4 *p* < 0.00002	0.5% (5/953)	1.9% (18/9530	Chi^2^ = 3.7 *p* < 0.2
	High school	1.5% (14/953)	16.9% (161/953)		8.9% (85/953)	9.4% (90/953)		2.4% (23/953)	15.9% (152/953)	
	University	13.7% (131/953)	65.5% (624/953)		56.0% (534/953)	23.2% (221/953)		15.2% (145/953)	64.0% (610/953)	
Marital status	Married	13.6% (130/953)	68.4% (652/953)	Chi^2^ = 3.2 *p* < 0.2	56.5% (538/953)	25.6% (244/953)	Chi^2^ = 15.9 *p* < 0.0004	14.9% (142/953)	67.2% (640/953)	Chi^2^ = 3.1 *p* < 0.2
	Cohabiting	1.8% (17/953)	14.3% (136/953)		8.7% (83/953)	7.3% (70/953)		2.6% (25/953)	13.4% (128/953)	
	Single parent & divorced	0.2% (2/953)	1.7% (16/953)		0.8% (8/953)	1.0% (10/953)		0.6% (6/953)	1.3% (12/953)	
Mode of delivery	Vaginal birth	8.8% (84/953)	47.1% (449/953)	Chi^2^ = 0.3 *p* < 0.9	35.3% (336/953)	20.7% (197/953)	Chi^2^ = 5.7 *p* < 0.06	9.9% (95/953)	46.0% (438/953)	Chi^2^ = 0.3 *p* < 0.9
	Elective c-section	3.8% (36/953)	19.2% (183/953)		15.2% (145/953)	5.9% (56/953)		4.1% (39/953)	17.0% (162/953)	
	Emergency c-section	3.0% (29/953)	18.0% (172/953)		15.5% (148/953)	7.5% (71/953)		4.1% (39/953)	18.9% (180/953)	
Maternal experience	0	1.9% (18/953)	7.8% (74/953)	Chi^2^ = 7.1 *p* < 0.2	7.0% (67/953)	2.6% (25/953)	Chi^2^ = 4.8 *p* < 0.4	2.1% (20/953)	7.6% (72/953)	Chi^2^ = 5.6 *p* < 0.3
	1	7.2% (69/953)	43.5% (415/953)		33.2% (316/953)	17.6% (168/953)		8.4% (80/953)	42.4% (404/953)	
	2	5.4% (51/953)	22,7% (216/953)		19.0% (181/953)	9.0% (86/953)		6.1% (58/953)	21.9% (209/953)	
	3	1.1% (11/953)	10.4% (99/953)		6.8% (65/953)	4.8% (45/953)		1.5% (15/953)	9.9% (95/953)	
Knowledge level regarding perinatal vaccination	Poor	3.4% (32/953)	40.9% (390/953)	Chi^2^ = 37.3 *p* < 0.0008	17.7% (169/953)	28.0% (267/953)	Chi^2^ = 266.7 *p* < 0.0002	5.4% (51/953)	40.4% (385/953)	Chi^2^ = 22.8 *p* < 0.00002
	Moderate	3.3% (31/953)	11.4% (109/953)		11.9% (113/0953)	2.0% (19/953)		3.5% (33/953)	10.4% (99/953)	
	Detailed	9.0% (86/953)	32.0% (305/953)		36.4% (347/953)	4.0% (38/953)		9.3% (89/953)	31.1% (296/953)	

c-section—caesarean section.

**Table 3:** Sociodemographic and obstetric variables and health-enhancing behaviors in relation to neonatal feeding strategy.

Data		Breastfeeding % (n/N)	Mixed Feeding % (n/N)	Formula Feeding % (n/N)	Chi^2^ Test *p*-Value
Age (years)	Mean ± SD Median (interquartile range 25–75%)	31.0 ± 4.4 31.0 (28.0–34.0)	31.1 ± 4.4 30.0 (28.0–34.0)	30.23 ± 4.6 31.00 (27.0–33.0)	Chi^2^ = 4.9 *p* < 0.29
	18–25	10.0% (76/764)	6.8% (7/103)	11.6% (10/86)	
	26–34	67.0% (512/764)	70.0% (72/103)	74.4% (64/86)	
	≥35	23.0% (176/764)	23.3% (24/103)	14.0% (12/86)	
Pre-pregnancy BMI, kg/m^2^	Mean ± SD Median (interquartile range 25–75%)	23.3 ± 4.1 22.5 (20.3–25.3)	23.7 ± 4.2 22.8 (20.8–26.0)	24.7 ± 5.6 22.7 (20.5–27.3)	Chi^2^ = 23.3 *p* < 0.0006
	Underweight (<18.5)	6.5% (50/764)	8.7% (9/103)	5.8% (5/86)	
	Normal weight (18.5–24.9)	67.1% (513/764)	60.2% (62/103)	57.0% (49/86)	
	Overweight (25–29.9)	7.8% (60/764)	20.4% (21/103)	14.0% (12/86)	
	Obesity (≥30)	18.5% (141/764)	10.7% (11/103)	23.3% (20/86)	
Residence	Urban, above 100,000 residents	48.2% (368/764)	54.4% (56/103)	41.9% (36/86)	Chi^2^ = 4.2 *p* < 0.65
	Urban, 10,000–100,000 residents	20.5% (157/764)	18.4% (19/103)	23.3% (20/86)	
	Urban, <10,000 residents	6.5% (50/764)	7.8% (8/103)	9.3% (8/86)	
	Rural	24.7% (189/764)	19.4% (20/103)	25.6% (22/86)	
Education	Vocational and primary	2.5% (19/764)	1.0% (1/103)	3.5% (3/86)	Chi^2^ = 6.4 *p* < 0.17
	High school	17.0% (130/764)	22.3% (23/103)	25.6% (22/86)	
	University	80.5% (615/764)	76.7% (79/103)	70.9% (61/86)	
Marital status	Married	82.9% (633/764)	80.6% (83/103)	76.7% (66/86)	Chi^2^ = 7.3 *p* < 0.12
	Cohabiting	15.3% (117/764)	15.5% (16/103)	23.3% (20/86)	
	Single parent & divorced	1.8% (14/764)	3.9% (4/103)	0% (0/86)	
Mode of delivery	Vaginal birth	57.6% (440/764)	50.0% (51/103)	48.8% (42/86)	Chi^2^ = 4. 6 *p* < 0.34
	Elective c-section	21.9% (167/764)	27.2% (28/103)	27.9% (24/86)	
	Emergency c-section	20.5% (157/764)	23.3% (24/103)	23.3% (20/86)	
Maternal experience	0	7.7% (59/764)	22.3% (23/103)	11.6% (10/86)	Chi^2^ = 30.1 *p* < 0.0001
	1	51.2% (391/764)	40.8% (42/103)	59.3% (51/86)	
	2	28.5% (218/764)	32.0% (33/103)	18.6% (16/86)	
	≥3	12.6% (96/764)	4.8% (5/103)	10.5% (9/86)	
Knowledge level regarding maternal vaccination	Poor	42.0% (321/764)	49.5% (51/103)	58.1% (50/86)	Chi^2^ = 11.0 *p* < 0.03
	Moderate	15.6% (119/764)	9.7% (10/103)	12.8% (11/86)	
	Detailed	42.4% (324/764)	40.8% (42/103)	29.1% (25/86)	
COVID-19 vaccination	Yes	66.5% (508/764)	67.0% (69/103)	60.5% (52/86)	Chi^2^ = 1.3 *p* < 0.53
	No	33.5% (256/764)	33.0% (34/103)	39.5% (34/86)	
Influenza vaccination	Yes	19.2% (147/764)	14.6% (15/103)	12.8% (11/86)	Chi^2^ = 3.2 *p* < 0.21
	No	80.8% (617/764)	85.4% (88/103)	87.2% (75/86)	
Dual vaccination	Yes	16.6% (127/764)	12.6% (13/103)	10.5% (9/86)	Chi^2^ = 3.0 *p* < 0.22
	No	83.4% (637/764)	87.4% (90/103)	89.5% (77/86)	

c-section—caesarean section.

**Table 4:** Sociodemographic and obstetric variables and health-enhancing behaviors in relation to maternal experience.

Data		No Maternal Experience % (n/N)	1 Child % (n/N)	2 Children % (n/N)	≥3 Children % (n/N)	Chi^2^ Test *p*-Value
Age (years)	Mean ± SD Median (interquartile range 25–75%)	30.6 ± 3.7 30.0 (28.0–32.0)	29.6 ± 4.1 29.0 (27.0–32.0)	32.1 ± 3.8 32.0 (29.0–35.0)	34.8 ± 4.5 36.0 (32.0–38.0)	
	18–25	5.4% (5/92)	15.1% (73/483)	4.1% (11/268)	3.6% (4/110)	Chi^2^ = 119.02 *p* < 0.0001
	26–34	80.4% (74/92)	71.8% (347/483)	67.5% (181/268)	41.8% (46/110)	
	≥35	14.1% (13/92)	13.3% (64/483)	28.0% (75/268)	54.5% (60/110)	
Pre-pregnancy BMI, kg/m^2^	Mean ± SD Median (interquartile range 25–75%)	23.8 ± 4.3 23.0 (20.7–25.7)	23.2 ± 4.2 22.2 (20.3–25.3)	23.6 ± 4.3 22.7 (20.6–25.3)	24.0 ± 4.8 23.5 (20.5–27.1)	
	Underweight (<18.5)	5.4% (5/92)	7.7% (37/483)	4.9% (13/268)	8.2% (9/110)	Chi^2^ = 6.25 *p* < 0.72
	Normal Weight (18.5–24.9)	64.1% (59/92)	66.3% (320/483)	67.5% (181/268)	58.2% (64/110)	
	Overweight (25–29.9)	19.6% (18/92)	17.2% (83/483)	17.5% (47/268)	23.6% (26/110)	
	Obesity (≥30)	10.9% (10/92)	9.1% (44/483)	9.7% (26/268)	10.0% (11/110)	
Residence	Urban, Above 100,000 Residents	62.0% (57/92)	50.9% (246/483)	44.4% (119/268)	34.5% (38/110)	Chi^2^ = 20.33 *p* < 0.006
	Urban, 10,000–100,000 Residents	15.2% (14/92)	18.6% (90/483)	21.2% (57/268)	31.8% (35/110)	
	Urban, <10,000 Residents	8.7% (8/92)	6.6% (32/483)	7.1% (19/268)	6.4% (7/110)	
	Rural	14.1% (13/92)	24.0% (116/483)	26.9% (72/268)	27.3% (30/110)	
Education	Vocational and Primary	0% (0/92)	1.9% (9/483)	3.0% (8/268)	5.5% (6/110)	Chi^2^ = 32.42 *p* < 0.0001
	High School	7.6% (7/92)	18.2% (88/483)	16.4% (44/268)	32.7% (36/110)	
	University	92.4% (85/92)	80.1% (387/483)	80.2% (215/268)	61.8% (68/110)	
Marital status	Married	82.6% (76/92)	78.1% (377/483)	86.6% (232/268)	88.2% (97/110)	Chi^2^ = 18.11 *p* < 0.008
	Cohabiting	17.4% (16/92)	19.0% (92/483)	12.7% (34/268)	10.0% (11/110)	
	Single Parent & Divorced	0% (0/92)	3.1% (15/483)	0.4% (1/268)	1.8% (2/110)	
Mode of delivery	Vaginal Birth	52.2% (48/92)	56.1% (271/483)	56.3% (151/268)	57.3% (63/110)	Chi^2^ = 53.04 *p* < 0.0001
	Elective C-Section	14.1% (13/92)	17.8% (86/483)	30.6% (82/268)	34.5% (38/110)	
	Emergency C-Section	33.7% (31/92)	26.3% (127/483)	12.7% (34/268)	8.2% (9/110)	
Knowledge level regarding maternal vaccination	Poor	42.4% (39/92)	43.3% (209/483)	43/7% (117/268)	51.8% (57/110)	Chi^2^ = 3.97 *p* < 0.68
	Moderate	15.2% (14/92)	15.7% (76/483)	13.1% (35/268)	13.6% (15/110)	
	Detailed	42.4% (39/92)	41.2% (199/483)	42.9% (115/268)	34.5% (38/110)	
COVID-19 vaccination	Yes	72.8% (67/92)	65.4% (316/483)	67.5% (181/268)	69.1% (65/110)	Chi^2^ = 4.75 *p* < 0.19
	No	27.2% (25/92)	34.8% (168/483)	32.1% (86/268)	40.9% (45/110)	
Influenza vaccination	Yes	21.7% (20/92)	16.6% (80/483)	21.6% (58/268)	13.6% (15/110)	Chi^2^ = 7.00 *p* < 0.07
	No	78.3% (72/92)	83.6% (404/483)	78.0% (209/268)	86.4% (95/110)	
Dual vaccination	Yes	19.6% (18/92)	14.3% (69/483)	19.0% (51/268)	10.0% (11/110)	Chi^2^ = 6.86 *p* < 0.08
	No	80.4% (74/92)	85.9% (415/483)	80.6% (216/268)	90.0% (99/110)	

c-section—caesarean section.

**Table 5:** Predictors of maternal vaccination.

Data	Odds Ratio (OR)	95% Lower—Upper Confidence Interval (CI)	*p*-Value
Age	1.07	1.02–1.11	0.005
BMI			
Normal Weight (18.5–24.9) (ref)			
Underweight (<18.5)	0.99	0.49–2.02	0.98
Overweight (25–29.9)	0.65	0.39–1.10	0.11
Obese (≥30)	0.34	0.15–0.77	0.009
Residence			
Urban, >100,000 Residents (ref)			
Urban, 10,000–100,000 Residents	0.52	0.31–0.87	0.02
Urban, <10,000 Residents	0.49	0.21–1.14	0.097
Rural	0.52	0.31–0.88	0.01
Education			
University (ref)			
High School	0.66	0.36–1.21	0.18
Vocational and Primary	1.77	0.56–5.73	0.33
Knowledge Level Regarding Maternal Vaccination			
High (ref)			
Moderate	1.05	0.64–1.70	0.86
Low	0.31	0.19–0.45	<0.001
Feeding Strategy			
Breastfeeding (ref)			
Mixed Feeding	0.878	0.41–1.48	0.45
Formula Feeding	0.87	0.41–1.84	0.72

**Table 6:** Predictors of breastfeeding among mothers.

Data	Odds Ratio (OR)	95% Lower—Upper Confidence Interval (CI)	*p*-Value
**BMI**			
Normal Weight (18.5–24.9) (ref)			
Underweight (<18.5)	0.77	0.41–1.45	0.42
Overweight (25–29.9)	0.94	0.61–1.46	0.80
Obese (≥30)	0.40	0.24–0.65	<0.001
**Maternal experience**			
1 child (ref)			
no maternal experience	0.41	0.25–0.67	<0.0001
2nd child	1.06	0.71–1.56	0.78
3rd and more child	1.76	0.96–3.26	0.07
**Level of Knowledge Regarding Perinatal Vaccination**			
High (ref)			
Moderate	1.15	0.67–1.97	0.62
Low	0.63	0.44–0.90	0.02
**Influenza Vaccination**			
Yes (ref)			
No	0.69	0.23–2.08	0.52
**Dual Vaccination**			
Yes (ref)			
No	1.15	0.35–3.77	0.82

## Data Availability

The data presented in this study are available on request from the corresponding author. The data are not publicly available due to privacy restrictions.

## Supplementary Materials

The following supporting information can be downloaded at: https://www.mdpi.com/article/10.3390/vaccines13040376/s1, Table S1: Results of Post Hoc Test of Multiple Comparisons with Bonferroni correction for significant outcomes: sociodemographic and health-enhancing behaviors of respondents in relation to maternal vaccination status; Table S2: Results of Post Hoc Test of Multiple Comparisons with Bonferroni correction for significant outcomes: sociodemographic and obstetric variables and health-enhancing behaviors in relation to neonatal feeding strategy; Table S3: Results of Post Hoc Test of Multiple Comparisons with Bonferroni correction for significant outcomes: Sociodemographic and obstetric variables and health-enhancing behaviors in relation to maternal experience.
