# Supplementary material for: Maternal Vaccination and Neonatal Feeding Strategies Among Polish Women
Source: Vaccines (Basel). 2025 Mar 31;13(4):376. doi: 10.3390/vaccines13040376 (PMC12031015; doi:10.3390/vaccines13040376)
Supplement: Supplementary file 1 [file vaccines-13-00376-s001.zip › vaccines-3478574-supplementary.pdf]

Supplementary Materials

Table S1. Results of Post Hoc Test of Multiple Comparisons with Bonferroni correction for significant outcomes: sociodemographic and health-enhancing behaviors of respondents in relation to maternal vaccination status.

Data	Categories compared	Dual Vaccination *p* - value	COVID-19 Vaccination *p* - value	Influenza Vaccination *p* - value
Age	18-25 vs 26-34	0.04	0.00004	
	18-25 vs ≥35	0.04	0.00002	n/a
	26-34 vs ≥35	1	0.9	
Pre-pregnancy BMI	Under vs Normal	1		1
	Under vs Overweight	1		0.66
	Under vs Obesity	0.42	n/a	0.41
	Normal vs Overweight	0.48		0.31
	Normal vs Obesity	0.12		0.11
	Overweight vs Obesity	1		1
Residence	>10,0000 vs 10,000-100,000	0.02	0.008	0.27
	>10,0000 vs <10,000	0.26	0.0004	1
	>10,0000 vs rural	0.0008	<0.000001	0.03
	10,000-100,000 vs <10000	1	0.62	1
	10,000-100,000 vs <rural	1	0.03	1
	<10,000 vs rural	1	1	1
Education	Vocational/primary vs high school	0.45	1	
	Vocational/primary vs university	1	0.02	n/a
	High school vs university	0.007	<0.00001	
Marital status	Married vs Cohabiting		0.002	
	Married vs Single/Divorced	n/a	0.09	n/a
	Cohabiting vs Single/Divorced		1	
Knowledge level regarding perinatal vaccination	Detailed vs Poor	<0.00001	<0.00001	0.0005
	Detaile vs Moderate	1	0.48	1
	Moderate vs Poor	<0.00001	<0.00001	0.0006

n/a - post hoc tests not applicable due to *p*>0.05 in overall test (Table 2)

Table S2. Results of Post Hoc Test of Multiple Comparisons with Bonferroni correction for significant outcomes: sociodemographic and obstetric variables and health-enhancing behaviors in relation to neonatal feeding strategy

Data	Categories compared	Neonatal feeding strategy

		p - value
Pre-pregnancy BMI	Under vs Normal	1
	Under vs Overweight	1
	Under vs Obesity	0.84
	Normal vs Overweight	0.002
	Normal vs Obesity	0.84
	Overweight vs Obesity	0.003
Maternal experience	0 vs 1	0.0002
	0 vs 2	0.02
	0 vs ≥3	0.012
	1 vs 2	0.22
	1vs ≥3	1
	2 vs ≥3	0.38
Knowledge level regarding perinatal vaccination	Detailed vs Poor	0.06
	Detaile vs Moderate	1
	Moderate vs Poor	0.25

post hoc tests applicable due to $p<0.05$ in overall test (Table 3)

Table S3. Results of Post Hoc Test of Multiple Comparisons with Bonferroni correction for significant outcomes: Sociodemographic and obstetric variables and health-enhancing behaviors in relation to maternal experience

Data	Categories compared	Maternal experience *p* - value
Age	18-25 vs 26-34	0.0004
	18-25 vs ≥35	<0.00001
	26-34 vs ≥35	<0.00001
Residence	>100,000 vs 10,000-100,000	0.005
	>100,000 vs <10,000	1
	>100,000 vs rural	0.042
	10,000-100,000 vs <10,000	1
	10,000-100,000 vs <rural	1
	<10000 vs rural	1
Education	Vocational/primary vs high school	1
	Vocational/primary vs university	0.048
	High school vs university	0.00005
Marital status	Married vs Cohabiting	0.09
	Married vs Single/Divorced	0.13
	Cohabiting vs Single/Divorced	0.39
Mode of delivery	vaginal birth vs elective c-section	0.01
	vaginal birth vs emergency c-section	0.00005
	elective c-section vs emergency c-section	<0.00001

c-section—caesarean section; post hoc tests applicable due to $p<0.05$ in overall test (Table 4)
